# PD-L1 Expression Is Associated With VEGFA and LADC Patients' Survival

**DOI:** 10.3389/fonc.2019.00189

**Published:** 2019-03-26

**Authors:** Shaochuan Liu, Tingting Qin, Yanan Jia, Kai Li

**Affiliations:** ^1^National Clinical Research Center for Cancer, Tianjin Medical University Cancer Institute and Hospital, Tianjin, China; ^2^Key Laboratory of Cancer Prevention and Therapy, Tianjin, China; ^3^Tianjin's Clinical Research Center for Cancer, Tianjin, China

**Keywords:** PD-L1, VEGFA, overall survival, progression-free survival, lung adenocarcinoma

## Abstract

**Objectives:** To elucidate the relationship between VEGFA and PD-L1 expression in lung adenocarcinoma (LADC).

**Methods:** PD-L1 and VEGFA expression were determined by immunohistochemistry with H-score on formalin-fixed paraffin-embedded resected LADC specimens of 129 cases.

**Results:** High PD-L1 expression in 53 (41.1%) patients, high VEGFA expression in 65 (50.4%), and co-expression in 18 (14.0%) were observed. Inverse correlation between expression of PD-L1 and VEGFA was found (*P* = 0.002, *r* = −0.274). VEGFA and PD-L1 expression were not significantly associated with the clinicopathological features. High PD-L1 expression was significantly association with all patients' poor progression-free survival and overall survival in a univariate analysis, but there was no significantly association with high VEGFA expression and prognosis. Co-expression of PD-L1 and VEGFA exhibited a worst overall survival compared to negative groups (*P* = 0.005).

**Conclusions:** These findings indicate that high PD-L1 expression could impact both poor overall survival and progression-free survival in patients with resected LADC. Co-expression of PD-L1 and VEGFA may be considered as an important prognostic factor for patients with resected lung adenocarcinoma.

## Introduction

Lung cancer is the most common and deadliest cancer worldwide (1). Non-small-cell lung cancer (NSCLC) accounts for approximately 80% of all patients, in which lung adenocarcinoma (LADC) is the most common. Although targeted drugs for driver gene mutations and immunotherapy in clinic have obtained a clinical benefit, prognosis for patients with LADC still remains unsatisfied (2). To improve the efficacy, it is of great importance to find ideal biomarkers to predict the progressive disease and overall survival of LADC patients.

In the last decade, inhibitors of PD-1 and its ligand PD-L1 were introduced into clinic. As a cell surface protein, PD-L1 is wildly expressed in variety of cells (such as immune cells, endothelial cells and tumor cells) and suppress immune-response lead by lymphocytes (3). Increased PD-L1 enhances tumor immune evasion and has been reported to be associated with a poor survival in various malignancies including lung cancer (4). With the development of anti-PD-1/PD-L1 inhibitors (such as nivolumab, pembrolizumab, ipilimumab), some clinical studies (5–8) have demonstrated excellent efficacy in patients with LADC, even in some with EGFR or ALK tyrosine kinase inhibitors (TKIs) resistance. Furthermore, it was found that high PD-L1 expression was closely correlated to a higher likelihood of objective response (OR) of anti-PD-1/PD-L1 in many studies (9, 10).

Like PD-L1, vascular endothelial growth factor (VEGF) is known as an immunosuppressive factor and plays an important role in tumor immune through promoting a tumor immunosuppressive microenvironment (11, 12). VEGFA, an important angiogenic factor in VEGF family, can exerts an important negative impact on immune cells' development and function (8). Some studies indicated that PD-L1 expression was significantly correlated with VEGF in clear cell renal carcinoma (13) and classical Hodgkin lymphoma (11). Additionally, over-expression of VEGFA has been confirmed in a majority of patients with NSCLC, some studies reported that there was a positive correlation between tumor vascularization and poor disease-free survival (DFS) or overall survival (OS) (14, 15).

Few studies reported that the connection between PD-L1 and VEGFA expression in patients with resected LADC. In the present study, we retrospectively reviewed on the data of patients with resected tissue of LADC and investigated the relationship between PD-L1 and VEGFA expression to further explore their potential efficacy-predictive value in anti-angiogenic therapy with immunotherapy and their prognostic significance.

## Materials and Methods

### Patients and Samples

We retrospectively reviewed 129 patients with LADC (56 with wild type, 56 with EGFR mutations, 13 with KRAS mutations, 4 with ALK mutations) surgically treated in Tianjin Medical University Cancer Institute and Hospital between December 2011 and September 2016. The postoperative follow-up period ranged from 21 to 79 months (median: 47 months). The patients' inclusion criteria included: (1) complete following-up date; (2) without neoadjuvant therapy before surgery; Regarding to preoperative staging assessment, all patients underwent positron emission tomography-computed tomography (PET-CT) or enhanced chest CT, contrast-enhanced computed tomography (ECT) and brain magnetic resonance imaging (MRI) if they were necessary. Twelve patients with stage IVA refer to incidental pleural metastases detected at surgery and 20 patients with stage III were found incidental N2 nodes during surgery. The clinical data included age at diagnosis, sex, smoking history, gene mutation status, histological subtypes, clinical stage, and postoperative chemotherapy; all parameters were retrieved based on electronic medical records. Progression-free survival (PFS) was defined as the interval between the date of resection and the date of disease progression or death. The follow-up for patients' survival with documented disease progression were censored on the last follow-up date. Overall survival (OS) was defined as the interval between the date of resection and either death due to any cause or the last follow-up. All clinical information was obtained from medical records. The study was approved by the Tianjin Medical University Cancer Institute and Hospital's Ethics Committee and informed consent was obtained from all patients in verbal form.

### Immunohistochemical Staining

Four micrometer thickness of tumor sections were deparaffinized and rehydrated. Antigen retrieval was performed at 130°C for 2 min, using EDTA solution (PH 11.0) for PD-L1, or citrate buffer (PH 6.0) for VEGFA. After blocking with 3% hydrogen peroxide and normal goat serum, the sections were incubated with primary antibody against PD-L1/CD274 antibody (66248-1-lg, Proteintech, USA) at dilution 1:1,200 or anti-VEGFA antibody (ab1316, Abcam, USA) at dilution 1:300 at 4°C overnight, followed by EIVISON plus (kit-9903, MXB, China), DAB kit (ZL1-9019, ZSGB-BIO, China) per the manufacturers protocol were used for coloration. Finally, the tumor sections were stained with hematoxylin and evaluated under a light-field microscope.

### Image Analysis

**Figure 1:**
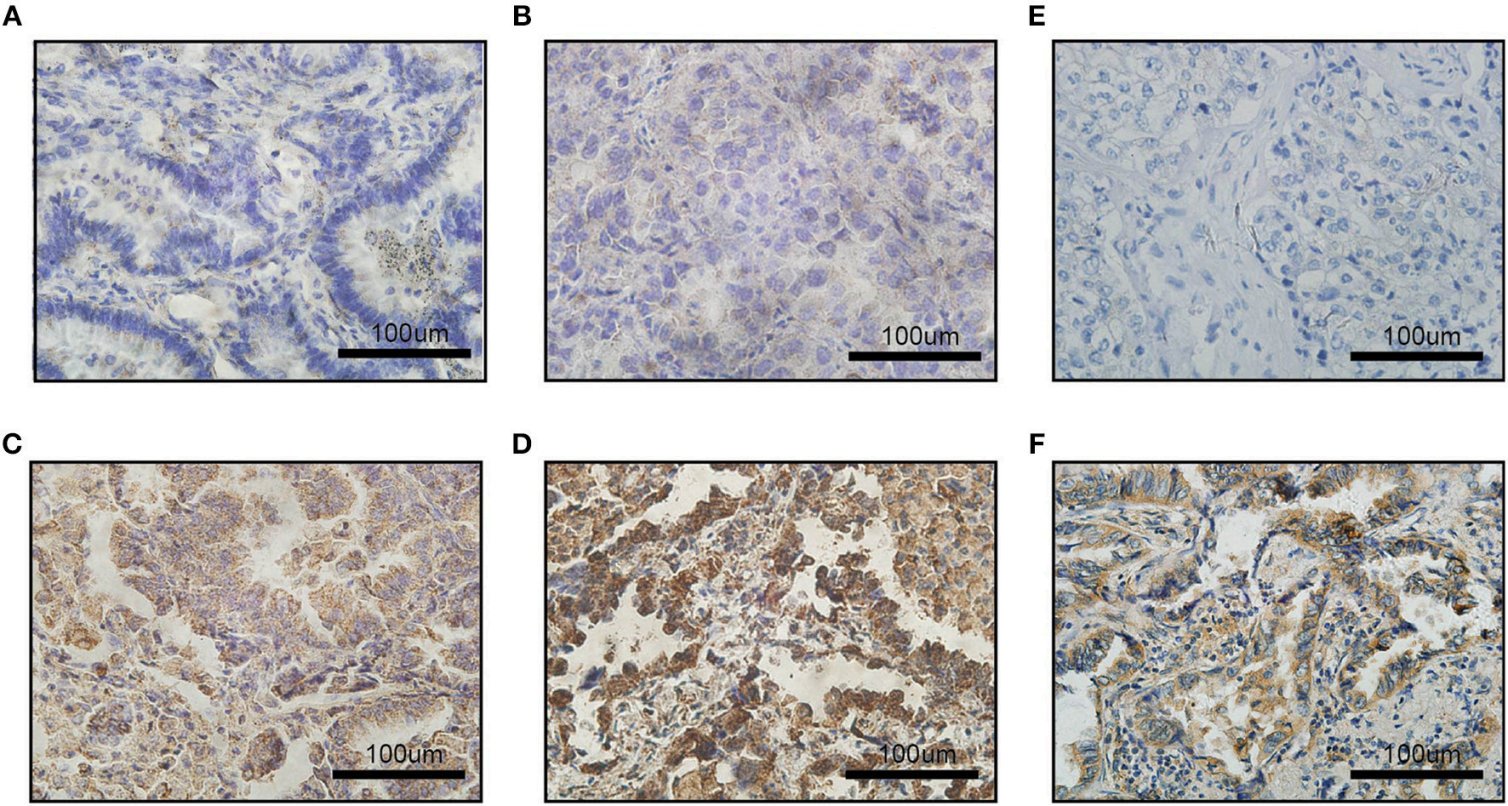
Images of immunohistochemical staining for PD-L1 and VEGFA expression in LADC. The staining strength of PD-L1 expression: **(A)** negative; **(B)** weak positive; **(C)** moderated positive; **(D)** strong positive; **(E)** VEGFA negative expression; **(F)** VEGFA positive expression; scale bar: 100 μm.

To measure the PD-L1 and VEGFA expression by IHC, stained sections were digitally analyzed at x400 resolution using an Olympus BX-UCB. PD-L1 and VEGFA expression were scored by H-score system. An H-score (range, 0–300) was calculated as the sum of the product of the highest intensity of staining (0, negative; 1, weak positive; 2, moderate positive; 3, strong positive) and percentage of tumor cells positive (0–100%; with any intensity of positive staining) (12). Two clinical pathologists assessed the intensity of the immunostaining on each section independently and in a blinded manner. According to previous literatures (16), we set the cut-off value at 100, i.e., H-score >100 as a PD-L1 overexpression group (generally 2+ intensity in >50% of tumor cells); when considering the VEGFA expression (17), we set the cut-off value at 50, i.e., H-score >50 as a VEGFA overexpression group (generally 2+ intensity in >25% of tumor cells) (Figure 1).

### Statistical Analysis

Statistical analyses were performed using SPSS v.21 (IBM Corp, Armonk, NY, USA) and survival curve was performed with GraphPad Prism 6 (USA, GraphPad Software). The relationships between PD-L1/VEGFA expression and clinical features were compared using Fisher's exact test; the linear relationship between PD-L1 and VEGFA expression was determined using Pearson's test. Patients' survival was estimated by the Kaplan-Meier method and compared using the log-rank test. Multivariate and univariate analysis were performed using the cox proportional hazards regression model to calculate the hazard ratio (HR) and 95% CI. Two-tailed *P* < 0.05 was considered statistically significant.

## Results

### Correlation of PD-L1 and VEGFA Expression With Clinic-Pathological Features

Baseline clinic-pathological characteristics of 129 LADC cases were summarized in Table 1. 61 (52.7%) patients were male; 25 (19.4%) patients were above age of 65; 57 (44.2%) patients were smokers; 92 (71.3%) patients were diagnosed as stage I/II; 41 (31.8%) cancer were characterized as acinar adenocarcinoma; high PD-L1 expression in 53 (41.1%) patients; high VEGFA expression in 65 (50.4%); 56 (43.4%) patients had EGFR mutations and 17 (12.2%) patients had other mutations including KRAS mutations and ALK mutations; 29 (38.2%) cases were in VEGFA-&PD-L1- group, 47 (61.8%) were in VEGFA+&PD-L1- group, 35 (66.0%) were in VEGFA-&PD-L1+ group and 18 (34.0%) were in VEGFA+&PD-L1+ group. No significant correlation of the PD-L1 or VEGFA expression and gender, clinical stage, age, histological subtypes, TNM, gene mutations, or smoking status were found (all *P* > 0.05) (Table 2).

**Table 1:** Clinical characteristics of 129 patients with LADC.

**Characteristics**	**ALL, *n* (%)**
**GENDER**	
Male	61 (52.7)
Female	68 (47.3)
**AGE**	
<65	104 (80.6)
≥65	25 (19.4)
**SMOKING HISTORY**	
Yes	57 (44.2)
No	72 (55.8)
**STAGE**	
I	73 (56.6)
II	19 (14.7)
III	25 (19.4)
IV	12 (9.3)
**HISTOLOGICAL SUBTYPES**	
Lepidic predomiant	47 (36.4)
Acinar predomiant	41 (31.8)
Papillary/micropapillary predomiant	23 (17.8)
Solid predomiant	7 (5.5)
Other	11 (8.5)
**T FACTOR**	
T1	72 (55.8)
T2	40 (31.0)
>T2	17 (13.2)
**N FACTOR**	
N0	86 (66.7)
N1	8 (6.2)
N2	35 (27.1)
**M FACTOR**	
M0	117 (90.7)
M1	12 (9.3)
**GENE STATUS**	
WT	56 (43.4)
EGFR	56 (43.4)
Other mutations	17 (13.2)
**VEGFA**	
H-socre ≤ 50	64 (49.6)
H-socre >50	65 (50.4)
**PD-L1**	
H-socre ≤ 100	76 (58.9)
H-socre >100	53 (41.1)
**POSTOPERATIVE THERAPY**	
Chemotherapy/chemoradiotherapy	46 (35.7)
Chemotherapy+TKI/TKI	7 (5.4)
Non-treatment	76 (58.9)

**Table 2:** Relationship between VEGFA/PD-L1 expression and the clinical characteristics in 129 patients with LADC.

**Characteristics**	**PD-L1 negative, *n* (%)**	**PD-L1 positive, *n* (%)**	***P*-value**	**VEGFA negative, *n* (%)**	**VEGFA positive, *n* (%)**	***P*-value**
**Gender**			0.703			0.250
Male	37 (48.7)	24 (45.3)		27 (42.2)	34 (52.3)	
Female	39 (51.3)	29 (54.7)		37 (57.8)	31 (47.7)	
**Age**			0.902			0.532
<65	61 (80.3)	43 (81.1)		53 (82.8)	51 (78.5)	
≥65	15 (19.7)	10 (18.9)		11 (17.2)	14 (21.5)	
**Smoking history**			0.383			0.690
Yes	36 (47.4)	21 (60.4)		27 (41.3)	30 (46.2)	
No	40 (52.6)	32 (39.6)		37 (58.7)	35 (53.8)	
**Stage**			0.867			0.733
I	45 (59.2)	28 (52.8)		35 (54.7)	38 (58.5)	
II	11 (14.5)	8 (15.2)		11 (17.2)	8 (12.3)	
III	13 (17.1)	12 (22.6)		11 (17.2)	14 (21.5)	
IV	7 (9.2)	5 (9.4)		7 (10.9)	5 (7.7)	
**Histological types**			0.529			0.350
Lepidic predominate	29 (38.1)	18 (34.0)		25 (39.1)	22 (33.8)	
Acinar predominate	26 (34.2)	15 (28.3)		15 (23.4)	26 (40.0)	
Papillary/micropapillary predominate	10 (13.2)	13 (24.5)		13 (20.3)	10 (15.4)	
Solid predominate	5 (6.6)	2 (3.8)		4 (6.3)	3 (4.6)	
Other	6 (7.9)	5 (9.4)		7 (10.9)	4 (6.2)	
**T**			0.051			0.281
T1	42 (55.3)	30 (56.6)		40 (62.5)	32 (49.2)	
T2	28 (36.8)	12 (22.7)		16 (25.0)	24 (37.0)	
>T2	6 (7.9)	11 (20.7)		8 (12.5)	9 (13.8)	
**N**			0.721			0.700
N0	50 (65.8)	36 (67.9)		43 (67.2)	43 (66.2)	
N1	6 (7.9)	2 (3.8)		5 (7.8)	3 (4.6)	
N2	20 (26.3)	15 (28.3)		16 (25.0)	19 (29.2)	
**M**			0.760			1.000
M0	68 (89.5)	49 (92.5)		58 (90.6)	59 (90.8)	
M1	8 (10.5)	4 (7.5)		6 (9.4)	6 (9.2)	
**Gene status**			0.081			0.417
WT	33 (43.4)	23 (43.4)		28 (43.8)	28 (43.1)	
EGFR	29 (38.2)	27 (50.9)		30 (46.9)	26 (40.0)	
Other mutations	14 (18.4)	3 (5.7)		6 (9.3)	11 (16.9)	
**Postoperative therapy**			0.693			0.472
Chemotherapy/chemoradiotherapy	28 (36.8)	18 (34.0)		22 (34.4)	24 (36.9)	
Chemotherapy+TKI/TKI	3 (3.9)	4 (7.5)		2 (3.1)	5 (7.7)	
Non-treatment	45 (59.3)	31 (58.5)		40 (62.5)	36 (55.4)	
**VEGFA**			**0.002**			
H-score ≤ 50	29 (38.2)	35 (66.0)				
H-score >50	47 (61.8)	18 (34.0)				

However, we found that expression of VEGFA was negatively correlated with expression of PD-L1 (*P* = 0.002, *r* = −0.274) (Supplement Figure 1).

### Correlation of PD-L1 and VEGFA Expression With Patients' Survival

**Figure 2:**
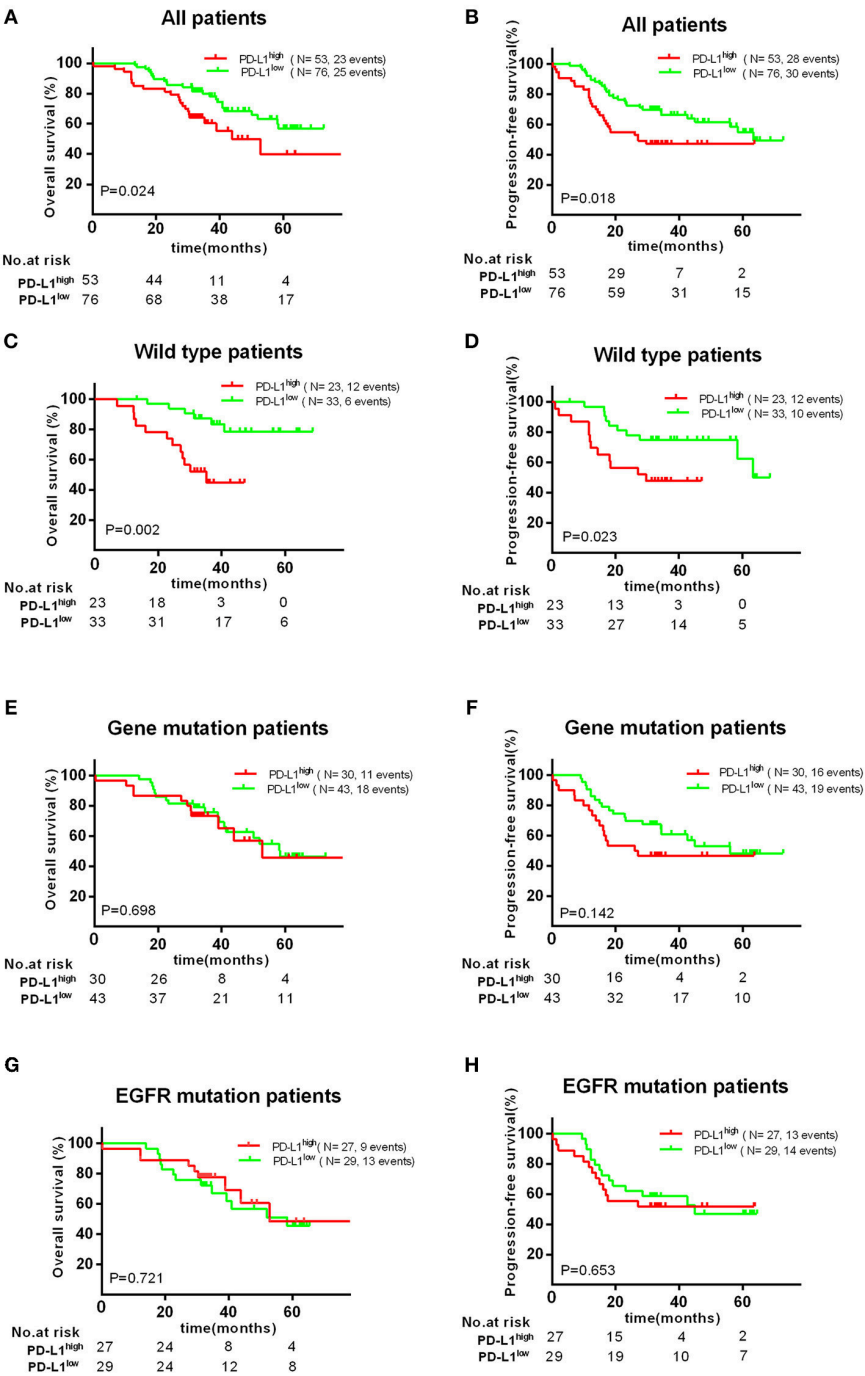
Association with PD-L1 expression and OS or PFS. There was a significant poor OS **(A)** and PFS **(B)** in PD-L1^high^ expression group. High PD-L1 expression has a significant unfavorable OS **(C)** and PFS **(D)** in patients with wild type, but no significant impact on patients' OS **(E,G)** and PFS **(F,H)** with EGFR mutations.

PD-L1 and VEGFA expression were measured in 129 patients wherein 53 patients (41.1%) had high expression PD-L1 (H-score >100) and 65 patients (50.4%) had high expression VEGFA (H-score >50). In all patients, PD-L1^high^ group showed a significant negative impact on the OS (*P* = 0.024) and PFS (*P* = 0.018) (Figure 2); however, high VEGFA expression was no significantly correlated with unfavorable OS (*P* = 0.190) and PFS (*P* = 0.325) in all patients (Supplement Figure 2).

**Figure 3:**
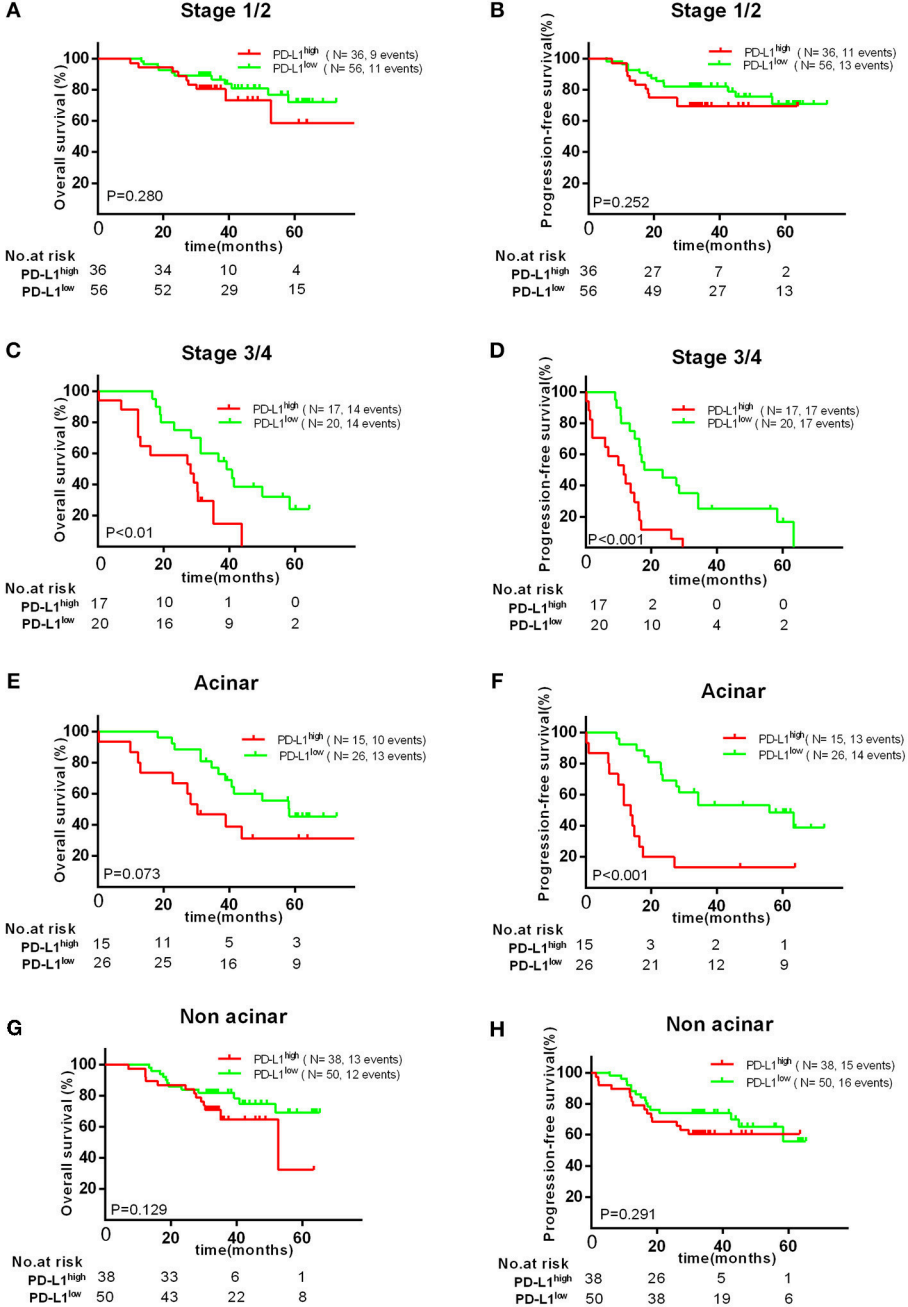
Kaplan–Meier curves showing OS **(A,C,E,G)** and PFS **(B,D,F,H)** of different subgroups (clinical stage I/II, III/IV, acinar, or non-acinar adenocarcinoma, respectively) with high and low expression of PD-L1.

Therefore, we further investigated the association between the expression of PD-L1 and survival in different subgroups. Firstly, we found high PD-L1 expression was not significantly correlated with adverse survival in patients of clinical stage I/II, in contrast, significantly correlated with poor OS (*P* < 0.010) and PFS (*P* < 0.001) in stage III/IV. Secondly, same result was found in patients with acinar adenocarcinoma, i.e., high PD-L1 expression was significantly correlated to an adverse PFS (*P* < 0.001), but not to poor OS (*P* = 0.129) or PFS (*P* = 0.291) in patients with non-acinar adenocarcinoma (Figure 3). Thirdly, we found high PD-L1 expression was no significantly correlated with OS (*P* = 0.698) and PFS (*P* = 0.142) in all gene mutations patients (EGFR, ALK, or KRAS). Because it was insufficient to analyze the relationship between PD-L1 expression and prognosis in only 17 (12.2%) patients with KRAS or ALK mutations, correlation between PD-L1 expression and survival in KRAS or ALK mutations group was not analyzed. However, we found that PD-L1 high expression has no significant impact on OS (*P* = 0.721) and PFS (*P* = 0.653) in patients with EGFR mutations; in wild type (WT) patients, PD-L1^high^ expression caused a poor OS (*P* < 0.010) and PFS (*P* = 0.023) compared to PD-L1^low^ cases (Figure 2).

**Figure 4:**
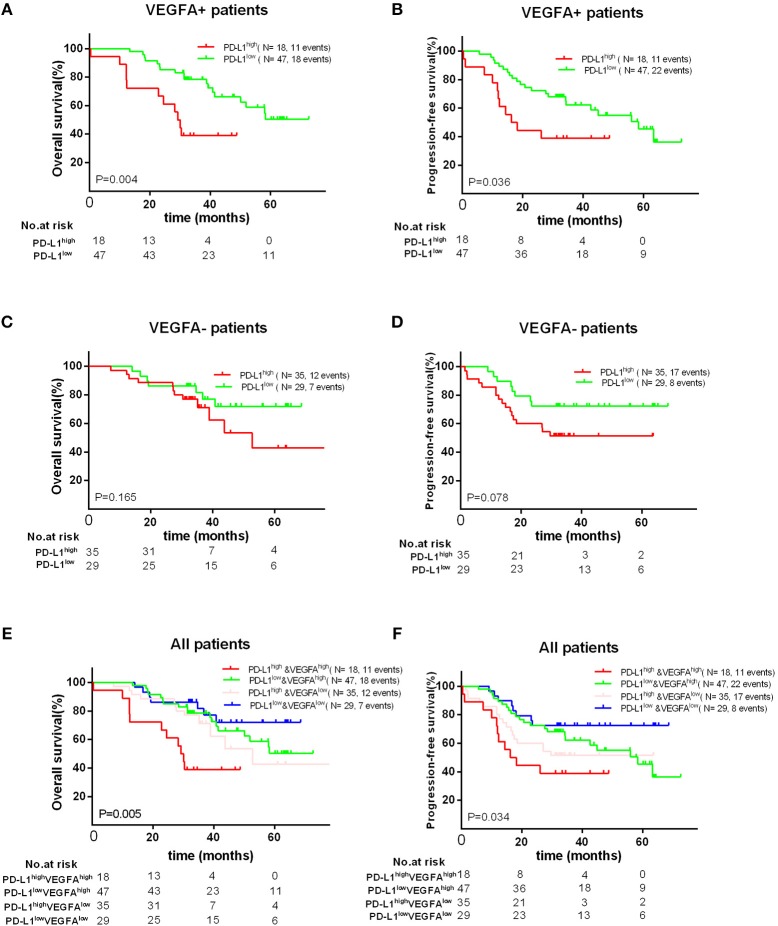
Kaplan–Meier curves showing OS **(A,C)** and PFS **(B,D)** of different groups (VEGFA+ or VEGFA-) with high and low of PD-L1 expression, showing OS **(E)** and PFS **(F)** in patients with PD-L1–&VEGFA–, PD-L1+&EVGFA–, PD-L1–&VEGFA+ and PD-L1+&VEGFA+ expression.

Finally, we found that high PD-L1 expression was as a poor prognostic role for PFS (*P* = 0.036) and OS (*P* = 0.004) in VEGFA+ group, but not a prognostic factor for OS (*P* = 0.165) and PFS (*P* = 0.078) in VEGFA- group. Concordantly, we found that VEGFA+&PD-L1+ group had the worst OS (*P* = 0.005) and PFS (*P* = 0.034) compared to other groups (VEGFA-&PD-L1-, VEGFA+&PD-L1-, or VEGFA-&PD-L1+) (Figure 4). Additionally, we failed to find a significant correlation between co-expression of PD-L1 and VEGFA and any clinical features (Supplement Tables 1, 2).

In a univariate analysis on the all patients, six clinical characteristics were determined as unfavorable prognostic factors for PFS: clinical stage III/IV [HR = 6.552 (95% CI 3.852–11.145), *P* < 0.0001], high PD-L1 expression [HR = 3.143 (95% CI 1.106–1.864), *P* = 0.019], advanced TNM (>T2; >N0; M1; all *P* < 0.001) and co-expression of PD-L1 and VEGFA [HR = 2.907 (95% CI 1.082–4.067), *P* = 0.028]. Those factors were also determined as unfavorable prognostic factors for OS. In a multivariate analysis, co-expression of VEGFA and PD-L1 was a poor prognostic factor for OS [HR = 3.230 (95% CI 1.388–7.518), *P* = 0.007] and high PD-L1 expression was an unfavorable prognostic factor for PFS [HR = 2.163 (95% CI 1.124–4.163, *P* = 0.021], but only clinical stage III/IV was adverse factor for both OS [HR = 2.657 (95% CI 1.085–6.504, *P* = 0.032] and PFS [HR = 4.064 (95% CI 1.815–9.097, *P* = 0.001] (Table 3).

**Table 3:** Univariate and multivariate cox analysis of factors for progression-free survival and overall survival in patients with LADC.

	**Overall survival**			**Progression-free survival**		
**Variable**	**HR**	**95% CI**	***P*-value**	**HR**	**95% CI**	***P*-value**
**UNIVARIATE COX ANALYSIS**						
Gender (female vs. male)	1.451	0.819–2.571	0.202	1.199	0.716–2.007	0.490
Age (<65 vs. ≥65)	1.461	0.744–2.870	0.271	1.065	0.634–1.790	0.812
Smoking history (no vs. yes)	1.286	0.729–2.267	0.385	1.046	0.543–2.018	0.892
T factor (T ≤ 2 vs. T >2)	3.735	1.997–6.985	**<0.0001**	3.778	2.073–6.855	**<0.001**
N factor (N0 vs. >N0)	2.798	1.580–4.955	**<0.0001**	3.674	2.178–6.194	**<0.001**
M factor (M0 vs. M1)	3.254	1.568–6.750	**0.002**	3.710	1.863–7.391	**<0.001**
Gene mutations (WT vs. EGFR)	1.099	0.662–1.942	0.745	1.277	0.761–2.141	0.354
Histological subtypes (acinar vs. non-acinar)	0.599	0.337–1.063	0.113	0.706	0.544–0.916	0.089
Stage (I/II vs. III/IV)	4.801	2.698–8.542	**<0.0001**	6.552	3.852–11.145	**<0.0001**
Postoperative therapy (else vs. adjuvant chemotherapy)	0.917	0.492–1.709	0.785	1.036	0.587–1.828	0.903
VEGFA expression (≤50 vs. >50)	1.470	0.823–2.624	0.193	1.297	0.711–2.183	0.327
PD-L1 expression (≤100 vs. >100)	1.924	1.080–3.427	**0.026**	3.143	1.106–1.864	**0.019**
Co-expression (else vs. PD-L1+/VEGFA+)	3.114	1.564–6.201	**0.001**	2.907	1.082–4.067	**0.028**
**MULTIVARIATE COX ANALYSIS**						
T factor	2.313	1.130–4.733	**0.022**	1.389	0.694–2.778	0.353
N factor	1.546	0.720–3.316	0.263	1.806	0.919–3.550	0.086
M factor	1.730	0.756–3.956	0.194	1.642	0.738–3.655	0.224
Stage	2.657	1.085–6.504	**0.032**	4.064	1.815–9.097	**0.001**
PD-L1 expression	1.494	0.735–3.036	0.267	2.163	1.124–4.163	**0.021**
Co-expression	3.230	1.388–7.518	**0.007**	1.360	0.621–2.980	0.443

*Statistically significant p-values (p < 0.05) are shown in bold*.

## Discussion

Recently, some clinical trials (6, 9) of PD-1/PD-L1 inhibitors have demonstrated durable clinical benefit in many patients. Several studies reported that the expression of PD-L1 is closely related to EGFR (18) mutations, KRAS (19) mutations, smoking history (19) and advanced tumor stage (18, 20), but we failed to define a significant correlation between PD-L1 expression and these clinical features in LADC, which is concordant with the results of other studies (20, 21). In addition, some studies suggested that VEGFA expression has a significant association with EGFR mutations, advanced clinical stage and lymph node metastasis (21). Similar to PD-L1 expression, in our patient cohort we didn't find a significant relationship between VEGFA expression and any clinical variables. Possibly due to the variety of cut-off value of PD-L1 and VEGFA positive expression in cancer, results of our study are different with that from previous some studies (positive PD-L1 ever defined as 1, 5, 10, or even 50%) (22, 23).

In the present study, we analyzed the correlation of PD-L1 and VEGFA expression and patients' prognosis. There was no significant correlation between VEGFA expression and survival found, as similar as the results in other studies (24, 25). But when considering PD-L1, we found a significant association between PD-L1 expression and prognosis. When analyzed in different subgroups, there was no significant association between PD-L1 expression and OS or PFS in patients with EGFR mutations; however, high expression PD-L1 has a significant correlation to poor OS and PFS in patients with wild type. These findings suggested that PD-L1 expression may be not a prognostic factor for survival in patients with EGFR mutations. A meta-analysis of three clinical trials (CheckMate057, POPLAR, and KEYNOTE-010) also found that patients with EGFR mutations did not represent a survival benefit from anti-PD-1/PD-L1 therapy compared with other treatment (26). Additionally, in patients with acinar adenocarcinoma and clinical stage III/IV, high expression of PD-L1 was associated with unfavorable OS and PFS, and other studies (27, 28) have found similar evidences.

The interesting finding from our study is that PD-L1 expression inversely related to the expression of VEGFA. About this issue, the traditional view is that VEGFA high expression will lead to vascular abnormalities, further aggravation of hypoxia and the activation of HIF-1a pathway in tumor tissues, which could result in increased expression of PD-L1 on tumor cells. According to this viewpoint there seems to be a close positive association between PD-L1 and VEGF expression in tumor tissues. Several studies also found that VEGF expression is positive associated with PD-L1 expression in clear cell renal cell carcinoma (13) and classical Hodgkin lymphoma (11). Nevertheless, other studies found that PD-L1 protein expression measured by IHC is inversely correlation with the mRNA expression of VEGFA in patients with clear cell renal cell carcinoma (29) and not associated with VEGF gene expression and patients' survival in angiosarcoma (30). In the previous study, no explanation of potential reason of such contradictory events and any report to observe the association among them in LADC were made. Here we suppose that a relatively normal vasculature is formed in tumor tissue with low expression of VEGFA (23, 31), which may lead to more immune cells infiltrating into tumor tissues, causing increased responding PD-L1 expression on tumor cells, therefore showing an opposite relationship between PD-L1 and VEGFA expression. Accordantly, some studies found that after blocking VEGFA, the expression of PD-L1 in tumor tissues will be upregulated in the preclinical model (32). Similarly, upregulation of PD-L1 has been found on both endothelial cells and tumor cells following treatment with anti-VEGFR2 therapy (33), which confirms the necessity of combination with anti-angiogenic and immunotherapy. It also explains from profile that a relationship between VEGFA and PD-L1 expression was not positively correlated. Of course, this is only an assumption and the underlying mechanisms need further to study.

Finally, we found that only 14.0% (18/129) patients revealed VEGFA+&PD-L1+, far fewer than the other types, but these patients had the worst prognosis compared to other groups. As far as we know, no study has reported this phenomenon in LADC, so we proposed another hypothesis: some LADC tumor cells in patients with co-expression of VEGFA and PD-L1 may have strong ability to evade from the attacks by immune cells, so even if few immune cells infiltrate into tumor tissue, through abnormal vasculature caused by high-expressed VEGFA, they can also cause “high-response” tumor cells to express high PD-L1. This explanation indicated that co-expression of PD-L1 and VEGFA may be as a predictor for high recurrent risk and poor prognosis. For these patients, the combination of anti-VEGF and anti-PDL1 could be an interesting treatment strategy. It also provides a theoretical possibility for screening optimal population to combination of anti-VEGFA and anti-PD-1/PD-L1 therapy. We also analyzed the relationship between co-expression of PD-L1 and VEGFA and clinical features, but failed to found a significant correlation between them and clinical features maybe due to only 18 patients with co-expression of PD-L1 and VEGFA.

Our study still has several limitations: 1. A similar limitation with other studies is the lack of standardized cut-off value of PD-L1 and VEGFA expression; 2. The amount of patient samples collected retrospectively were relatively small; 3. It should be emphasized that it is the initial and immature study to explore the correlation of VEGFA and PD-L1 expression in LADC; we demonstrated the correlation between them, but the underlying mechanisms is still unclear.

## Conclusion

High expression PD-L1 is a poor factor on PFS and OS in patients with WT, clinical stage III/IV or acinar adenocarcinoma, but has no significant impact on patients with early stage and EGFR mutations. Expression of VEGFA is negatively correlated with the expression of PD-L1, but patients with co-expression of PD-L1 and VEGF will lead to significantly poor prognosis than negative ones. Our study also provides the theoretical possibility to screen optimal population of combination with anti-PD-L1/PD-1 and anti-VEGF therapy in LADC.

## Author Contributions

SL: design of study and manuscript preparation; YJ: data statistics; TQ and KL: critical revision of the manuscript.

### Conflict of Interest Statement

The authors declare that the research was conducted in the absence of any commercial or financial relationships that could be construed as a potential conflict of interest.

## Supplementary Material

Supplement Figure 1 Scatter diagram showing the correlation of VEGFA expression and PD-L1 expression based on the results of H-score.

Supplement Figure 2 Kaplan–Meier curves showing OS **(A)** and PFS **(B)** of all patients with high and low of VEGFA expression.

Supplement Table 1 Correlation of expression of VEGFA and/or PD-L1 and the clinical characteristics in 129 patients with LADC.

Supplement Table 2 Correlation of co-expression of VEGFA and PD-L1 and the clinical characteristics in 129 patients with LADC.

## References

[1] Bray F Ferlay J Soerjomataram I Siegel RL Torre LA Jemal A. Cancer statistics 2018: GLOBOCAN estimates of incidence and mortality worldwide for 36 cancers in 185 countries. CA Cancer J Clin. (2018) 68:394–424. 10.3322/caac.21492 30207593

[2] Ettinger DS Wood DE Aisner DL Akerley W Bauman J Chirieac LR. Non-small cell lung cancer, version 5.2017, NCCN clinical practice guidelines in oncology. J Nat Comprehens Cancer Netw. (2017) 15:504–35. 10.6004/jnccn.2017.0050 28404761

[3] Blank C Gajewski TF Mackensen A. Interaction of PD-L1 on tumor cells with PD-1 on tumor-specific T cells as a mechanism of immune evasion: implications for tumor immunotherapy. Cancer Immunol Immunother. (2005) 54:307–14. 10.1007/s00262-004-0593-x 15599732 PMC11032914

[4] Patel SP Kurzrock R. PD-L1 expression as a predictive biomarker in cancer immunotherapy. Mol Cancer Ther. (2015) 14:847–56. 10.1158/1535-7163.MCT-14-0983 25695955

[5] Qiao M Jiang T Ren S Zhou C. Combination strategies on the basis of immune checkpoint inhibitors in non-small-cell lung cancer: where do we stand? Clin Lung Cancer. (2018) 19:1–11. 10.1016/j.cllc.2017.06.005 28716463

[6] Herbst RS Baas P Kim DW Felip E Pérez-Gracia JL Han JY. Pembrolizumab versus docetaxel for previously treated, PD-L1-positive, advanced non-small-cell lung cancer (KEYNOTE-010): a randomised controlled trial. Lancet. (2016) 387:1540–50. 10.1016/s0140-6736(15)01281-7 26712084

[7] Reck M Rodriguez-Abreu D Robinson AG Hui R Csoszi T Fulop A. Pembrolizumab versus chemotherapy for PD-L1-positive non-small-cell lung cancer. N Engl J Med. (2016) 375:1823–33. 10.1056/NEJMoa1606774 27718847

[8] Huang Y Goel S Duda DG Fukumura D Jain RK. Vascular normalization as an emerging strategy to enhance cancer immunotherapy. Cancer Res. (2013) 73:2943–8. 10.1158/0008-5472.CAN-12-4354 23440426 PMC3655127

[9] Herbst RS Soria JC Kowanetz M Fine GD Hamid O Gordon MS. Predictive correlates of response to the anti-PD-L1 antibody MPDL3280A in cancer patients. Nature. (2014) 515:563–7. 10.1038/nature14011 25428504 PMC4836193

[10] Tumeh PC Harview CL Yearley JH Shintaku IP Taylor EJ Robert L. PD-1 blockade induces responses by inhibiting adaptive immune resistance. Nature. (2014) 515:568–71. 10.1038/nature13954 25428505 PMC4246418

[11] Koh YW Han JH Yoon DH Suh C Huh J. PD-L1 expression correlates with VEGF and microvessel density in patients with uniformly treated classical Hodgkin lymphoma. Ann Hematol. (2017) 96:1883–90. 10.1007/s00277-017-3115-6 28842748

[12] Howitt BE Sun HH Roemer MG Kelley A Chapuy B Aviki E. Genetic basis for PD-L1 expression in squamous cell carcinomas of the cervix and vulva. JAMA Oncol. (2016) 2:518–22. 10.1001/jamaoncol.2015.6326 26913631

[13] Shin SJ Jeon YK Kim PJ Cho YM Koh J Chung DH. Clinicopathologic analysis of PD-L1 and PD-L2 expression in renal cell carcinoma: association with oncogenic proteins status. Ann Surg Oncol. (2016) 23:694–702. 10.1245/s10434-015-4903-7 26464193

[14] Zhu CQ Shih W Ling CH Tsao MS. Immunohistochemical markers of prognosis in non-small cell lung cancer: a review and proposal for a multiphase approach to marker evaluation. J Clin Pathol. (2006) 59:790–800. 10.1136/jcp.2005.031351 16873561 PMC1860456

[15] Salgia R. Prognostic significance of angiogenesis and angiogenic growth factors in nonsmall cell lung cancer. Cancer. (2011) 117:3889–99. 10.1002/cncr.25935 21858799 PMC3160199

[16] Yang CY Lin MW Chang YL Wu CT Yang PC. Programmed cell death-ligand 1 expression is associated with a favourable immune microenvironment and better overall survival in stage I pulmonary squamous cell carcinoma. Eur J Cancer. (2016) 57:91–103. 10.1016/j.ejca.2015.12.033 26901614

[17] Liontos M Trigka EA Korkolopoulou P Tzannis K Lainakis G Koutsoukos K. Expression and prognostic significance of VEGF and mTOR pathway proteins in metastatic renal cell carcinoma patients: a prognostic immunohistochemical profile for kidney cancer patients. World J Urol. (2017) 35:411–9. 10.1007/s00345-016-1890-7 27395374

[18] Chen YY Wang LB Zhu HL Li XY Zhu YP Yin YL. Relationship between programmed death-ligand 1 and clinicopathological characteristics in non-small cell lung cancer patients. Chin Med Sci J. (2013) 28:147–51. 10.1016/s1001-9294(13)60040-1 24074616

[19] Calles A Liao X Sholl LM Rodig SJ Freeman GJ Butaney M. Expression of PD-1 and its ligands, PD-L1 and PD-L2, in smokers and never smokers with KRAS-mutant lung cancer. J Thorac Oncol. (2015) 10:1726–35. 10.1097/JTO.0000000000000687 26473645

[20] Yuan XH Yang J Wang XY Zhang XL Qin TT Li K. Association between EGFR/KRAS mutation and expression of VEGFA, VEGFR and VEGFR2 in lung adenocarcinoma. Oncol Lett. (2018) 16:2105–12. 10.3892/ol.2018.8901 30008907 PMC6036498

[21] Reinmuth N Jauch A Xu EC Muley T Granzow M Hoffmann H. Correlation of EGFR mutations with chromosomal alterations and expression of EGFR, ErbB3 and VEGF in tumor samples of lung adenocarcinoma patients. Lung cancer. (2008) 62:193–201. 10.1016/j.lungcan.2008.03.011 18450321

[22] Garon EB Rizvi NA Hui R Leighl N Balmanoukian AS Eder JP. Pembrolizumab for the treatment of non-small-cell lung cancer. N Engl J Med. (2015) 372:2018–28. 10.1056/NEJMoa1501824 25891174

[23] Horn L Spigel DR Vokes EE Holgado E Ready N Steins M. Nivolumab versus docetaxel in previously treated patients with advanced non-small-cell lung cancer: two-year outcomes from two randomized, open-label, phase III trials (CheckMate 017 and CheckMate 057). J Clin Oncol. (2017) 35:3924–33. 10.1200/jco.2017.74.3062 29023213 PMC6075826

[24] Berardi R Brunelli A Pagliaretta S Paolucci V Conti A Goteri G. Impact of VEGF, VEGFR, PDGFR, HIF and ERCC1 gene polymorphisms on thymic malignancies outcome after thymectomy. Oncotarget. (2015) 6: 19305–15. 10.18632/oncotarget.4191 26254278 PMC4662492

[25] Ramanathan R Olex AL Dozmorov M Bear HD Fernandez LJ Takabe K. Angiopoietin pathway gene expression associated with poor breast cancer survival. Breast Cancer Res Treat. (2017) 162:191–8. 10.1007/s10549-017-4102-2 28062977 PMC5290093

[26] Lee CK Man J Lord S Links M Gebski V Mok T. Checkpoint inhibitors in metastatic EGFR-mutated non-small cell lung cancer-a meta-analysis. J Thorac Oncol. (2017) 12:403–7. 10.1016/j.jtho.2016.10.007 27765535

[27] Zhang Y Wang L Li Y Pan Y Wang R Hu H. Protein expression of programmed death 1 ligand 1 and ligand 2 independently predict poor prognosis in surgically resected lung adenocarcinoma. Onco Targets Ther. (2014) 7:567–73. 10.2147/OTT.S59959 24748806 PMC3990506

[28] Nakamura Y Kobayashi T Nishii Y Suzuki Y Saiki H Ito K. Comparable immunoreactivity rates of PD-L1 in archival and recent specimens from non-small cell lung cancer. Thorac Cancer. (2018) 9:1476–82. 10.1111/1759-7714.12861 30209885 PMC6209791

[29] Joseph RW Parasramka M Eckel-Passow JE Serie D Wu K Jiang L. Inverse association between programmed death ligand 1 and genes in the VEGF pathway in primary clear cell renal cell carcinoma. Cancer Immunol Res. (2013) 1:378–85. 10.1158/2326-6066.CIR-13-0042 24778130 PMC4322777

[30] Bagaria SP Gatalica Z Maney T Serie D Parasramka M Attia S. Association between programmed death-ligand 1 expression and the vascular endothelial growth factor pathway in angiosarcoma. Front Oncol. (2018) 8:71. 10.3389/fonc.2018.00071 29623256 PMC5874284

[31] Maenhout SK Thielemans K Aerts JL. Location, location, location: functional and phenotypic heterogeneity between tumor-infiltrating and non-infiltrating myeloid-derived suppressor cells. Oncoimmunology. (2014) 3:e956579. 10.4161/21624011.2014.956579 25941577 PMC4292540

[32] Schmittnaegel M Rigamonti N Kadioglu E Cassará A Wyser Rmili C Kiialainen A. Dual angiopoietin-2 and VEGFA inhibition elicits antitumor immunity that is enhanced by PD-1 checkpoint blockade. Sci Transl Med. (2017) 9:eaak9670. 10.1126/scitranslmed.aak9670 28404865

[33] Allen E Jabouille A Rivera LB Lodewijckx I Missiaen R Steri V. Combined antiangiogenic and anti-PD-L1 therapy stimulates tumor immunity through HEV formation. Sci Transl Med. (2017) 9:eaak9679. 10.1126/scitranslmed.aak9679 28404866 PMC5554432

