# Supplementary figures and images for: PD-L1 Expression Is Associated With VEGFA and LADC Patients' Survival
Source: Front Oncol. 2019 Mar 26;9:189. doi: 10.3389/fonc.2019.00189 (PMC6443993; doi:10.3389/fonc.2019.00189)

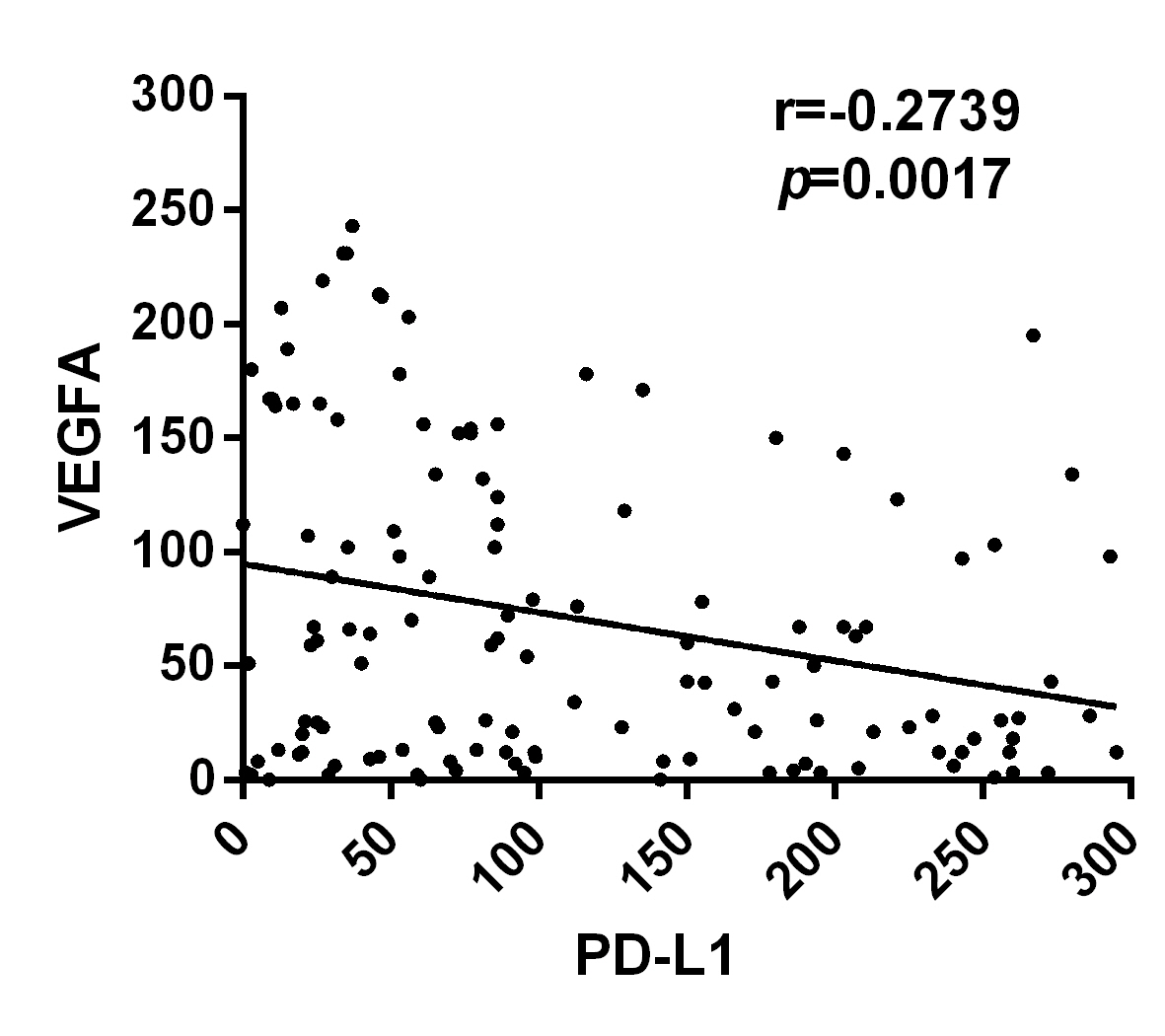

Supplement: Supplement Figure 1 — Scatter diagram showing the correlation of VEGFA expression and PD-L1 expression based on the results of H-score. [file Image_1.JPEG]

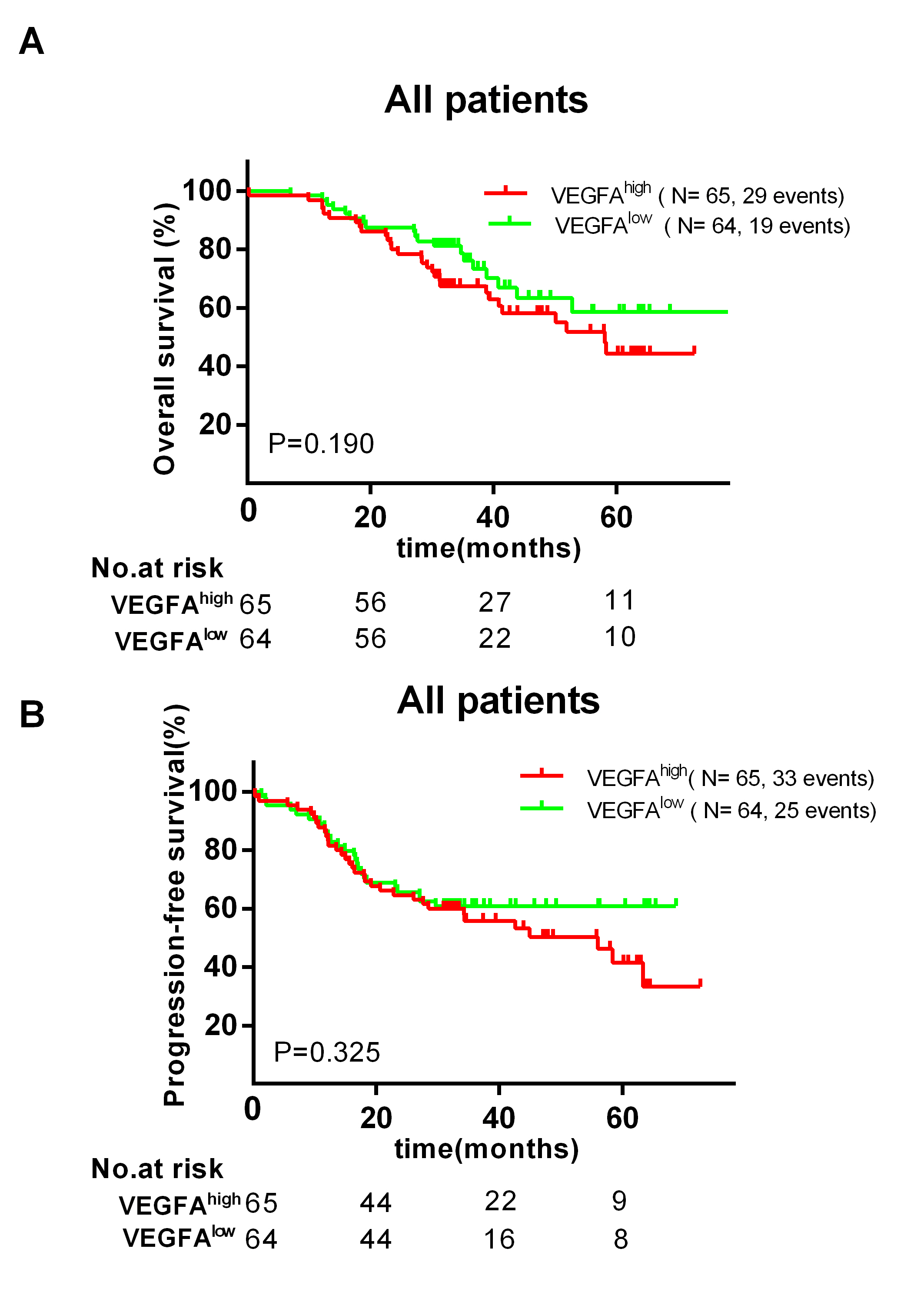

Supplement: Supplement Figure 2 — Kaplan–Meier curves showing OS (A) and PFS (B) of all patients with high and low of VEGFA expression. [file Image_2.JPEG]
