# Supplementary material for: PD-L1 Expression Is Associated With VEGFA and LADC Patients' Survival
Source: Front Oncol. 2019 Mar 26;9:189. doi: 10.3389/fonc.2019.00189 (PMC6443993; doi:10.3389/fonc.2019.00189)
Supplement: Supplement Table 1 — Correlation of expression of VEGFA and/or PD-L1 and the clinical characteristics in 129 patients with LADC. [file Table_1.DOC]

**Supplement table 1. Correlation of expression of VEGFA and/or PD-L1 and the clinical characteristics in 129 patients with LADC.**

|  | **VEGFA-/PD-L1-, n (%)** | **VEGFA+/PD-L1-, n (%)** | **VEGFA-/PD-L1+, n (%)** | **VEGFA+/PD-L1+, n (%)** | **P -value** |
| --- | --- | --- | --- | --- | --- |
| **T factor** |  |  |  |  | 0.146 |
| T2≤ | 28(96.6) | 42(89.4) | 28(73.7) | 14(77.8) |  |
| ＞T2 | 1(3.4) | 5(10.6) | 7(26.3) | 4(22.2) |  |
| **N factor** |  |  |  |  | 0.918 |
| N0 | 20(69.0) | 30(63.8) | 23(65.7) | 13(72.2) |  |
| >N0 | 9(31.0) | 17(36.2) | 12(34.3) | 5(27.8) |  |
| **M factor** |  |  |  |  | 0.722 |
| M0 | 25(86.2) | 43(91.5) | 33(94.3) | 16(88.9) |  |
| >M0 | 4(13.8) | 4(8.5) | 2(5.7) | 2(11.1) |  |
| **Clinical stage** |  |  |  |  | 0.888 |
| Ⅰ/Ⅱ | 22(75.9) | 34(72.3) | 24(64.9) | 12(66.7) |  |
| Ⅲ/Ⅳ | 7(24.1) | 13(27.7) | 11(35.1) | 6(33.3) |  |
| **Gender** |  |  |  |  | 0.409 |
| Male | 14(48.3) | 23(48.9) | 13(37.1) | 11(61.1) |  |
| Female | 15(51.7) | 24(51.1) | 22(62.9) | 7(38.9) |  |
| **Age** |  |  |  |  | 0.762 |
| ＜65 | 25(86.2) | 36(76.6) | 28(80.0) | 15(83.3) |  |
| ≥65 | 4(13.8) | 11(23.4) | 7(20.0) | 3(16.7) |  |
| **Smoking history** |  |  |  |  | 0.510 |
| Yes | 15(51.7) | 21(44.7) | 12(34.3) | 9(50.0) |  |
| No | 14(48.3) | 26(55.3) | 23(65.7) | 9(50.0) |  |
| **Acianr adenocarcinoma** |  |  |  |  | 0.189 |
| Yes | 6(20.7) | 20(42.6) | 9(25.7) | 6(33.3) |  |
| No | 23(79.3) | 27(57.4) | 26(74.3) | 12(66.7) |  |
| **EGFR mutations** |  |  |  |  | 0.054 |
| Yes | 8(27.6) | 21(44.6) | 21(60.0) | 6(33.3) |  |
| No | 21(72.4) | 26(55.4) | 14(40.0) | 12(66.7) |  |

**Supplement table 2. Correlation of co-expression of VEGFA and PD-L1 and the clinical characteristics in 129 patients with LADC**

|  | **Without PD-L1+/VEGFA+, n (%)** | **PD-L1+/VEGFA+, n (%)** | **P-value** | **PD-L1-/VEGFA -, n (%)** | **PD-L1+/VEGFA+ , n (%)** | **P-value** |
| --- | --- | --- | --- | --- | --- | --- |
| **T factor** |  |  | 0.257 |  |  | 0.063 |
| T2≤ | 98 (88.2) | 14 (77.8) |  | 28 (96.6) | 14 (77.8) |  |
| ＞T2 | 13 (11.8) | 4 (22.2) |  | 1 (3.4) | 4 (22.2) |  |
| **N factor** |  |  | 0.590 |  |  | 0.812 |
| N0 | 73 (65.8) | 13 (72.2) |  | 20 (69.0) | 13 (72.2) |  |
| >N0 | 38 (34.2) | 5 (27.8) |  | 9 (31.0) | 5 (27.8) |  |
| **M factor** |  |  | 1.000 |  |  | 1.000 |
| M0 | 101 (91.0) | 16 (88.9) |  | 25 (86.2) | 16 (88.9) |  |
| >M0 | 10 (9.0) | 2 (11.1) |  | 4 (13.8) | 2 (11.1) |  |
| **Clinical stage** |  |  | 0.638 |  |  | 0.521 |
| Ⅰ/Ⅱ | 80 (72.1) | 12 (66.7) |  | 22 (75.9) | 12 (66.7) |  |
| Ⅲ/Ⅳ | 31 (18.9) | 6 (33.3) |  | 7 (24.1) | 6 (33.3) |  |
| **Gender** |  |  | 0.205 |  |  | 0.391 |
| Male | 50 (45.0) | 11 (61.1) |  | 14 (48.3) | 11 (61.1) |  |
| Female | 61 (55.0) | 7 (38.9) |  | 15 (51.7) | 7 (38.9) |  |
| **Age** |  |  | 1.000 |  |  | 1.000 |
| ＜65 | 89 (80.2) | 15 (83.3) |  | 25 (86.2) | 15 (83.3) |  |
| ≥65 | 22 (19.8) | 3 (16.7) |  | 4 (13.8) | 3 (16.7) |  |
| **Smoking history** |  |  | 0.592 |  |  | 0.908 |
| Yes | 48 (43.2) | 9 (50.0) |  | 15 (51.7) | 9 (50.0) |  |
| No | 63 (56.8) | 9 (50.0) |  | 14 (48.3) | 9 (50.0) |  |
| **EGFR mutations** |  |  | 0.352 |  |  | 0.675 |
| Yes | 50 (45.0) | 6 (33.3) |  | 8 (27.6) | 6 (33.3) |  |
| No | 61 (55.0) | 12 (66.7) |  | 21 (72.4) | 12 (66.7) |  |
| **Acinar adenocarcinoma** |  |  | 0.879 |  |  | 0.493 |
| Yes | 35 (31.5) | 6 (33.3) |  | 6 (20.7) | 6 (33.3) |  |
| No | 76 (68.5) | 12 (66.7) |  | 23 (79.3) | 12 (66.7) |  |
